# The impact of retirement on inpatient healthcare utilization in Guangzhou, China: a regression discontinuity analysis of 189,031 health insurance claims

**DOI:** 10.1186/s12877-021-02664-2

**Published:** 2022-04-29

**Authors:** Xintong Zhao, Yuehua Liu, Xin Zhang, Till Bärnighausen, Simiao Chen

**Affiliations:** 1 School of Labor and Human Resources, Renmin University of China, Beijing, China; 2 Vanke School of Public Health, Tsinghua University, Beijing, China; 3 School of Statistics, Beijing Normal University, Beijing, China; 4 Heidelberg Institute of Global Health, Heidelberg Medical School, Heidelberg University, Heidelberg, Germany; 5 Chinese Academy of Medical Sciences & Peking Union Medical College, Beijing, China

**Keywords:** Retirement, inpatient, healthcare utilization, fuzzy regression discontinuity design, China

## Abstract

**Background:**

Previous studies suggest that retirement, a major life event, affects overall healthcare utilization. We examine, the effects of retirement on inpatient healthcare utilization, including effect heterogeneity by gender, disease category, and type of health service.

**Methods:**

We used routine health insurance claims data (*N* = 87,087) spanning the period 2021 - September 2013 from the Urban Employee Basic Medical Insurance (UEBMI), a mandatory social health insurance for working and retired employees in urban China. We applied a non-parametric fuzzy regression discontinuity design using the statutory retirement age in urban China as an exogenous instrument to measure the causal effect of retirement on six measures of inpatient healthcare utilization.

**Results:**

Retirement reduced total hospital costs (-84.71 Chinese Yuan (CNY), 95% confidence interval (CI) -172.03 – 2.61), shortened length of hospital stays (-44.59, 95% CI -70.50 – -18.68), and increased hospital readmissions (0.06, 95% CI 0.00 – 0.12) and primary hospital visits (0.06, 95% CI 0.02 – 0.09) among women. Retirement did not significantly change inpatient healthcare utilization among men. The retirement effects among women varied by disease category. Specifically, retirement substantially increased hospitalizations for non-communicable diseases (NCDs), yet had only modest or no effect on hospitalizations for communicable diseases or injuries. Retirement effects among women also varied by the type of services. For relatively inexpensive services, such as nonoperative treatment, there were surges in the extensive margin (hospital readmission). For relatively expensive and invasive services, such as surgeries, retirement reduced the intensive margin (out-of-pocket expenditures and length of stay).

**Conclusions:**

Retirement decreases overall use of inpatient healthcare for women. The examination on the disease-related heterogeneous effects helps with the introduction and implementation of integrated healthcare delivery and appropriate incentive schemes to encourage better use of healthcare resources among older adults.

**Supplementary Information:**

The online version contains supplementary material available at 10.1186/s12877-021-02664-2.

## Background

Retirement is a major life event that can affect health [1–3], yet its impact on healthcare utilization and expenditures is not well understood. Several studies report increases in healthcare utilization after retirement [4, 5], whereas other studies report negative effects on outpatient care utilization [6], hospitalization [7], and healthcare expenditures on the extensive margin [8], predominantly in developed countries. One previous study investigated the causal effects of retirement on healthcare utilization in China [9]; it reported a general increase in healthcare utilization after retirement. In this study, we estimate the retirement effects on inpatient healthcare utilization in a new dataset in one important geographical context in China, in Guangzhou, one of China's major industrial and most populous cities. We also determine whether the causal effect of retirement on healthcare utilization differ across disease categories and types of healthcare services. As rising health expenditures due to population aging have brought fiscal challenges to healthcare systems – and nearly 70% of the total health spending in China is on hospitalization – a deeper understanding of retirement effects on inpatient healthcare utilization may provide valuable insights when formulating policies aimed at improving the use of healthcare resources and the health of older populations.

Two important mechanisms could explain the divergent findings on the effect of retirement on healthcare utilization. The first may be differences in disease-specific inpatient healthcare utilization [10]. For example, converging evidence shows that retirement is more likely to induce changes in non-communicable disease (NCD) related health outcomes, such as weight [3], mental health [1], and health behaviors [6]. These changes could influence NCD-related hospitalizations. In contrast, inpatient healthcare demand for time-critical treatment due to communicable diseases or injuries might be relatively unaffected by changes in working status or income level. This important heterogeneity according to disease type has been largely neglected in the empirical literature and inference based exclusively on average treatment effects may be misleading [11]. Second, income and substitution effects may also contribute to the variation in the demand for health services at retirement. Health care utilization may drop because of the sharp decline in income following retirement [12]. The other one is the substitution effect due to healthcare price changes following retirement. In particular, retirement decreases the opportunity cost of time [4, 5] and typically increases the reimbursement rate [13], which may increase healthcare utilization. These two competing effects render the net effect of retirement on healthcare utilization an inconclusive empirical issue.

This paper contributes to the existing literature on the causal effects of retirement on healthcare utilization in several ways. First, taking advantages of a novel and large dataset of hospital discharge records from the Urban Employee Basic Medical Insurance (UEBMI) in China, we investigated, for the first time, the heterogenous causal effects of retirement on healthcare utilization and expenditures by gender, disease category, and type of health service. The macroeconomic burden of NCDs is equivalent to an annual tax of 3.42% on the economy in China [14], while the average growth rate of total health expenditures has been outpacing the annual rate of gross domestic product growth for the past decade. These facts give urgency to understanding the effects of major life events on different types of healthcare utilization. Our findings may be used by policy makers improve the allocation of resources in the health system and prevent the underuse of healthcare among older adults.

Second, by focusing on a wide range of objective outcome measures, our results provide insights into both the income and substitution effects of retirement. Specifically, we used administrative health insurance claims data on individual total inpatient cost, out-of-pocket expenditures, hospital readmission, length of stay, and hospital level of care to quantify the effects of retirement on inpatient healthcare utilization. We thus elaborate and test the canonical model of demand for health services developed by Feldstein, which proposed income and price as key determinants of healthcare utilization [15].

Third, we employed a quasi-experimental approach, a non-parametric fuzzy regression discontinuity design, to estimate causally strong effect sizes. We make use of the fact that the probability of retiring increases discontinuously at the statutory retirement age, which is 60 years for male workers and 50 years for female workers in China. These thresholds have been quite strictly enforced in urban enterprises, public institutions, social organizations, and individual economic organizations since the 1980 s, and have been used in the previous literature as a source of exogenous variation in retirement [16–18]. We use this regression discontinuity approach to examine the causal effects of retirement on inpatient healthcare utilization in China.

## Methods

### Data source

We used data from the administrative health insurance claims dataset of the UEBMI in Guangzhou, the capital city of Guangdong province, China. The UEBMI was established in 1998 and provides almost universal health insurance coverage for employees in urban sectors. The cost-sharing rate for inpatient admission is 10-20% for workers, and 7-14% for retirees, respectively. The dataset is a population-based retrospective cross-sectional survey that collects individual-level administrative data for participants covered by UEBMI. It includes information on medical claims and demographic characteristics (date of birth and sex). Each claim is recorded as a separate entry and includes information on hospitalization date, length of stay, hospital level, hospital diagnosis, medical expenses and retirement status. There are three hospital levels in China, all open to patients without appointment. The hospital discharge diagnoses are coded into 7,228 health conditions in accordance with the International Classification of Disease 10th revision (ICD-10) in the database. We mapped the ICD-10 codes to the disease categories used in the Global Burden of Disease study [19].

We enrolled all medical claims submitted for inpatient care for around 418,000 participants from all hospitals in Guangzhou, between 1 January 2012, and 30 September 2013. We restricted the sample to participants aged between 40 (480 months) and 75 (900 months). We then excluded the self-employed or those in flexible employment. We included only people with a local urban hukou, thereby dropping records of admitted rural residents or people from other cities than Guangzhou. We further excluded claims in which the hospital stay extended beyond the sample period. Finally, we excluded records with missing information on healthcare utilization as well as individuals with a length of hospital stay exceeding 90 days. Our final sample included 87,087 participants with 189,031 inpatient medical claims over 21 months.

### Variables

For the empirical analyses, we constructed the following 6 outcome variables to measure inpatient healthcare utilization: (1) *Total inpatient cost*: average monthly total hospital expenditures (which reflect the overall use of healthcare). (2) *Out-of-pocket expenditures*: average monthly out-of-pocket hospital expenditures. (3) *Hospital readmission*: a dummy variable indicating hospital readmission within one year of discharge. We use 1-year readmission instead of 30-day or 90-day readmission to capture participants’ individual health choices and behaviors beyond the control of hospital. (4) *Average length of stay*: average number of days a patient spent in a hospital. (5) *Primary hospital visit ratio*: proportion of all hospital visits that were visits to primary hospitals, ranging from 0 to 1. (6) *Tertiary hospital visit ratio*: proportion of all hospital visits that were visits to tertiary hospitals, ranging from 0 to 1. Changes in out-of-pocket expenditures and length of stay are intensive margin effects on healthcare utilization, and changes in hospital readmission are extensive margin effects. All outcome variables were measured at the individual level during the 21-month study period.

The key independent variable we examine is retirement. The existing literature defines retirement in several ways, including receipt of pension, self-reported retirement status, and exit from the labor market [6, 20]. We assume that retirement affects healthcare utilization through changes in time and financial constraints. Thus, we employed “registered retirement” to measure retirement; that is, we coded individuals as retired in the UEBMI if they had registered with their local social security department for retirement after they had completed the retirement procedures, because people are eligible for pension benefits once they register their retirement.

**Fig. 1:**
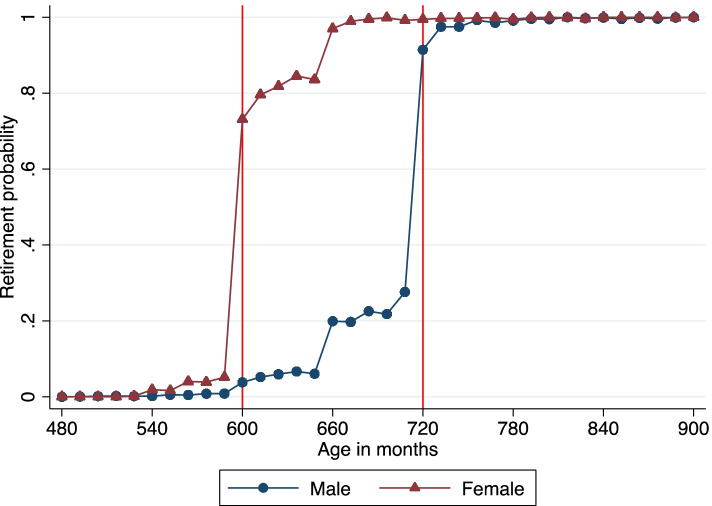
Retirement Probability by Age and Gender in the Urban Formal Sector. NOTE: The vertical lines at ages 60 years (720 months) for men and 50 years (600 months) for women are the statutory retirement ages

Figure 1 displays the retirement probability in the urban formal sector by age and gender. The retirement probability rises steadily prior to age 60 years (720 months) for men and 50 years (600 months) for women and then jumps sharply at these statutory retirement ages. By age 75 years, the retirement probability for both genders is approximately 1. The statutory retirement ages are 50 years for female workers and 55 years for female civil servants. Since our sample includes only information on urban formal sector employees, no civil servants are included. Thus, we use 50 years as the statutory retirement age for female workers. This decision is also consistent with previous studies [9].

### Study design

We used a regression discontinuity design to establish the causal effects of retirement on inpatient healthcare utilization. The regression discontinuity design is a quasi-experimental study design that can be used when an institutional threshold rule is applied to assign an intervention, policy, or treatment. In a sharp regression discontinuity design, the probability of being treated deterministically jumps from 0 to 1 at the threshold; in a fuzzy regression discontinuity design, the probability of receiving the treatment on one side of the threshold is significantly higher than on the other side, but this discontinuity is not deterministic. Under some weak assumptions, it is possible to infer the causal effect of the treatment by capturing the difference in outcome variables across the assignment threshold [21, 22].

The assignment variable for our regression discontinuity analysis is age. The thresholds used in the analysis are the age cutoff points: 720 months for men and 600 months for women. Although these statutory retirement ages apply to most workers, treatment is not completely determined by age. Early retirement is allowed for those in poor health or engaged in high-risk, high-intensity work. Thus, we employed a fuzzy regression discontinuity design using the retirement discontinuity at the statutory retirement ages as instruments to measure the causal effects of retirement on healthcare utilization. We focus on the following parameter to estimate the causal effects of retirement:

$${\tau }_{FRD}=\frac{\underset{x\downarrow a}{\text{lim}}\mathbb{E}\left[{H}_{i}|{X}_{i}=x\right]-\underset{x\uparrow a}{\text{lim}}\mathbb{E}\left[{H}_{i}|{X}_{i}=x\right]}{\underset{x\downarrow a}{\text{lim}}\mathbb{E}\left[{R}_{i}|{X}_{i}=x\right]-\underset{x\uparrow a}{\text{lim}}\mathbb{E}\left[{R}_{i}|{X}_{i}=x\right]}$$

where ${H}_{i}$ is one of the 6 measures of individual *i*’s healthcare utilization. ${X}_{i}$ is the age in months. ${R}_{i}$ is the dummy variable for retirement and *a* is the statutory retirement age (720 months for men and 600 months for women). ${\tau }_{FRD}$ is the local average treatment effect at the threshold, that is, the average difference in healthcare utilization for those who retired at the statutory retirement ages. We estimated this parameter separately for men and women.

In the empirical analyses, we conducted nonparametric fuzzy regression discontinuity analyses to avoid assuming any specific distribution function of the assignment variable. We fitted regression discontinuity models using local linear regression to avoid data overfitting [23]. We used a triangular kernel function to construct the local-polynomial estimators, which put more weight on observations closely above and below the cutoff point. We employed the optimal bandwidth method [24] to determine the bandwidth around the age threshold.

After estimating the main effects, we further measured changes in inpatient healthcare utilization for specific disease categories using the method above. We assume that the elasticity of demand for healthcare varies across people with different diseases and types of health services. Accordingly, we divided all the health conditions into three disease categories using the discharge diagnosis [25]: (1) communicable, maternal, neonatal, and nutritional diseases; (2) NCDs; (3) injuries. We also divided the treatment of conditions into surgical treatment and non-operative treatment based on the type of service. We then fitted regression discontinuity models across these subgroups in the heterogeneity analysis.

To check the validity of the fuzzy regression discontinuity design, we performed several sensitivity analyses. These include estimation with a restricted sample (10 years below and above the age cutoff point) and estimation based on a variety of potential bandwidths. We also conducted a series of placebo tests to check whether outcomes were discontinuous at ages other than the statutory retirement ages. The 95% confidence intervals are reported in the tables. All statistical analyses were performed using Stata version 14.0.

## Results

### Sample characteristics

Table 1 summarizes the sample characteristics. Column 1, 3 and 5 present the mean and standard deviation (SD) of outcome variables at ages 58-59 years for men, and 48-49 years for women. We identified 87,087 inpatients aged between 40 and 75 years between January 2012 and September 2013. About 48% of the inpatients were women. The 1-year hospital readmission rate was 27%. Monthly average total inpatient cost was 604 Chinese Yuan (CNY), which equals US$ 96 at an exchange rate of 1 US$ = 6.3 CNY. One quarter of this cost was out-of-pocket expenditures. The average length of stay was 10 days. The visit ratio was 8% and 74% for primary hospitals and tertiary hospitals, respectively. Of all discharge diagnoses, 7% were communicable, maternal, neonatal, and nutritional diseases, 88% were NCDs and 5% were injuries.

**Table 1:** Summary Statistics

	Mean (SD) or N (%)				
	Full sample	Male (*n* = 45,139)		Female (*n* = 41,948)	
	(*n* = 87,087)	Full	Age 58-59	Full	Age 48-49
*Outcome Variables*					
Total Inpatient Cost	601.49 (781.81)	656.99 (906.2)	661.18 (906.93)	541.78 (615.16)	522.77 (540.08)
Out-of-pocket Expenditures	160.51 (238.5)	178.4 (297.71)	187.93 (242.54)	141.26 (148.34)	164.87 (153.59)
Hospital Readmission	0.27 (0.44)	0.27 (0.44)	0.28 (0.46)	0.26 (0.44)	0.16 (0.52)
Average Length of Stay	10.28 (7.6)	10.63 (8.02)	10.7 (7.98)	9.9 (7.11)	9.49 (6.69)
Primary Hospital Visit Ratio	0.07 (0.24)	0.07 (0.24)	0.06 (0.21)	0.08 (0.25)	0.05 (0.22)
Tertiary Hospital Visit Ratio	0.74 (0.42)	0.74 (0.41)	0.76 (0.4)	0.74 (0.42)	0.77 (0.41)
*Treatment Variable*					
Retirement	48810 (56.05%)	21075 (46.69%)	-	27735 (66.12%)	-
*Discharge Diagnoses*					-
Communicable, Maternal, Neonatal, and Nutritional Diseases	5946 (6.83%)	2903 (6.43%)	-	3051 (7.27%)	-
Non-communicable Diseases	76946 (88.36%)	39790 (88.15%)	-	37156 (88.58%)	-
Injuries	4187 (4.81%)	2446 (5.42%)	-	1741 (4.15%)	-
*Types of Services*					
Surgery	40775 (46.82%)	20874 (46.24%)	-	19901 (47.44%)	-
Non-surgery	46312 (53.18%)	24264 (53.76%)	-	22048 (52.56%)	-
*Other Variables*					-
Age in Months	686.94 (123.15)	692.67 (123.37)	-	680.77 (122.61)	-

SD = standard deviation

### Manipulation of assignment variable

It is required in the regression discontinuity design that the assignment variable (age in months) must not be manipulated around the threshold. We checked for manipulation visually using a histogram of the continuous assignment variable (Additional file 1: Fig. S1). We found no visual evidence of heaping or concentration around the thresholds for both genders, suggesting that the assignment variable was not manipulated. This is not surprising because the UEBMI claims data were collected by hospital staff who had no incentives and likely little means to manipulate the age data.

### Causal effect estimates

Table 2 shows results from the discontinuity regression estimates of the causal effect of retirement. We report both conventional estimates with conventional variance estimators and the bias-corrected estimates with robust variance estimators. The latter increases the finite-sample variability of the t-statistic [26]. In general, the estimates indicate that retirement has significant instantaneous effects on healthcare utilization for women, but not for men. Female inpatients spent less, had shorter length of hospital stay, and were more likely to have a subsequent hospital stay after retirement. For example, the drop in total inpatient cost was estimated to be 97 CNY (95% CI = -203.38 – 9.69) per month, which was 18.7% (18% SD) of the monthly total inpatient cost among women two years prior to the statutory retirement age. In addition, we found that primary hospitals, rather than the higher level hospitals, became more common for female inpatients after retirement: there was a significant increase in the primary hospital visit ratio for women, whereas the tertiary hospital visit ratio did not change significantly.

**Table 2:** Regression Discontinuity Estimates of the Effects of Retirement on Inpatient Healthcare Utilization

	(1)	(2)	(3)	(4)	(5)	(6)
	Total inpatient cost	Out-of-pocket expenditures	Hospital readmission	Average length of stay	Primary hospital visit ratio	Tertiary hospital visit ratio
*Panel A: Full sample*						
Conventional	9.75 (-55.13 - 74.63)	-28.81*** (-46.62 - -11.00)	0.03 (-0.01 - 0.06)	-0.20 (-0.86 - 0.47)	0.03*** (0.02 - 0.05)	0.02 (-0.02 - 0.06)
Robust	19.86 (-58.70 - 98.42)	-24.95** (-46.67 - -3.22)	0.03 (-0.02 - 0.07)	-0.23 (-1.03 - 0.57)	0.03*** (0.01 - 0.05)	0.02 (-0.03 - 0.07)
*Panel B: Male*						
Conventional	70.13 (-49.37 - 189.62)	-17.98 (-51.7 - 15.79)	-0.04 (-0.10 - 0.02)	0.13 (-0.86 - 1.13)	0.02 (-0.01 - 0.06)	0.02 (-0.02 - 0.06)
Robust	102.99 (-35.81 - 241.80)	-9.34 (-48.63 - 29.94)	-0.04 (-0.11 - 0.04)	0.22 (-0.99 - 1.42)	0.02 (-0.01 - 0.06)	0.04 (-0.02 - 0.09)
*Panel C: Female*						
Conventional	-84.71* (-172.03 - 2.61)	-44.59*** (-70.50 - -18.68)	0.06** (0.00 - 0.12)	-0.85* (-1.73 - 0.03)	0.06*** (0.02 - 0.09)	0.00 (-0.06 - 0.06)
Robust	-96.85* (-203.38 - 9.69)	-46.68*** (-78.43 - -14.93)	0.07* (-0.00 - 0.14)	-0.99* (-2.03 - 0.06)	0.06*** (0.02 - 0.10)	0.00 (-0.07 - 0.07)

NOTE: In each panel, entries in the first two rows are the conventional regression discontinuity estimates with 95% confidence intervals, entries in the third and fourth rows are the bias-corrected estimates with 95% confidence intervals, and entries in the fifth row are the optimal bandwidth. 95% confidence intervals are in parentheses. *** p < 0.01, ** p < 0.05, * p < 0.1

### Heterogenous effects by disease category and type of service

Table 3 shows the regression discontinuity estimates by disease category and type of treatment. The pattern of gender differences was consistent with the main results. Panel A suggests that most of the change in inpatient healthcare utilization at retirement for women is driven by NCD hospitalizations, the most common reason for hospitalizations for the elderly. For NCD-related hospitalizations, the drop in total inpatient cost, out-of-pocket expenditures, and average length of stay for this subgroup were 110 CNY (95% CI = -222.2 – 1.67), 50 CNY (95%CI = -84.03 – -16.01), and 1.4 days (95% CI = -2.62 – -0.29), respectively; the increase in hospital readmissions and the primary hospital visit ratio were 0.09 (95% CI = 0.01 – 0.16) and 0.05 (95% CI = 0.01 – 0.09), respectively. For communicable disease-related hospitalizations, there was no evidence of a change in healthcare utilization, except for a drop in out-of-pocket expenditures and a rise in the primary hospital visit ratio. For injury-related hospitalizations, the increased total inpatient cost was associated with a longer average length of stay.

**Table 3:** Regression discontinuity Estimates by Discharge Diagnosis and Type of Service

		(1)	(2)	(3)	(4)	(5)	(6)
		Total inpatient cost	Out-of-pocket expenditures	Hospital readmission	Average length of stay	Primary hospital visit ratio	Tertiary hospital visit ratio
*Panel A: By Disease Category*							
1. Communicable diseases	Male	20.46 (-270.4 - 311.4)	-29.01 (-92.26 - 34.24)	-0.04 (-0.25 - 0.18)	0.75 (-2.23 - 3.73)	0.01 (-0.13 - 0.14)	-0.11 (-0.33 - 0.10)
	Female	-107.1 (-258.0 - 43.76)	-45.75** (-84.06 - -7.44)	-0.01 (-0.24 - 0.22)	1.28 (-1.90 - 4.47)	0.17* (-0.03 - 0.37)	-0.14 (-0.45 - 0.18)
2. Non-communicable diseases	Male	103.9 (-42.84 - 250.5)	-12.78 (-53.93 - 28.36)	-0.04 (-0.12 - 0.04)	0.02 (-1.25 - 1.29)	0.02 (-0.02 - 0.06)	0.04 (-0.02 - 0.11)
	Female	-110.2* (-222.2 - 1.67)	-50.02*** (-84.03 - -16.01)	0.09** (0.01 - 0.16)	-1.45** (-2.62 - -0.29)	0.05** (0.01 - 0.09)	-0.01 (-0.07 - 0.06)
3. Injuries	Male	724.5 (-180.9 - 1,630)	35.52 (-67.16 - 138.2)	-0.05 (-0.32 - 0.22)	4.43 (-2.92 - 11.78)	0.04 (-0.20 - 0.28)	0.21 (-0.14 - 0.56)
	Female	477.7* (-35.00 - 990.4)	47.40 (-95.52 - 190.3)	-0.13 (-0.46 - 0.20)	8.39* (-0.13 - 16.9)	-0.10 (-0.41 - 0.20)	0.32 (-0.15 - 0.79)
*Panel B: By Type of Service*							
4. Surgery	Male	143.5 (-99.86 - 386.9)	-22.66 (-83.69 - 38.37)	-0.08** (-0.15 - -0.02)	0.50 (-1.23 - 2.23)	0.03 (-0.01 - 0.07)	0.04 (-0.04 - 0.1)
	Female	-189.4*** (-314.1 - -64.74)	-69.10*** (-106.5 - -31.65)	0.04 (-0.04 - 0.12)	-1.65** (-3.04 - -0.27)	0.01 (-0.03 - 0.03)	0.00 (-0.09 - 0.09)
5. Non-surgery	Male	41.78 (-42.61 - 126.2)	-13.74 (-43.64 - 16.15)	-0.03 (-0.12 - 0.05)	-0.21 (-1.59 - 1.17)	0.03 (-0.01 - 0.07)	0.04 (-0.05 - 0.13)
	Female	77.79 (-41.74 - 197.3)	-5.18 (-35.84 - 25.47)	0.11 (-0.02 - 0.24)	-0.04 (-1.67 - 1.59)	0.11*** (0.04 - 0.19)	-0.02 (-0.15 - 0.12)

NOTE: Entries are the regression discontinuity estimates with robust variance estimators.95% confidence intervals are in parentheses.*** p < 0.01, ** p < 0.05, * p < 0.1

Panel B in Table 3 shows different consequences of retirement across types of services for women. For surgical treatment, out-of-pocket expenditures and average length of stay dropped by 189 CNY (95% CI =-314.1 – -64.74 ), 69 CNY (95% CI =-106.5 – -31.65), and 1.7 days (95% CI = -3.04 – -0.27). For non-operative treatment, there was an increase in the primary hospital visit ratio (0.11, 95% CI = 0.04 – 0.19).

### Robustness checks and placebo test

We tested the robustness of our findings by restricting the sample to 10 years below and above the age cutoff. The estimated discontinuities did not change (Additional file 1: Table S1). The drop in total inpatient cost, out-of-pocket expenditures and average length of stay for women were 103 CNY (95% CI = -217.3 – 12.0), 48 CNY (95% CI = -81.86 – -14.03), 0.98 days (95% CI =-2.00 – 0.05), respectively; the increase in hospital readmission and primary hospital visit ratio were 0.07 (95% CI =0.00 – 0.143) and 0.06 (95% CI =0.02 – 0.11), respectively. Results were also similar when using bandwidths of 12, 18, 24, 48, and 72 months (Additional file 1: Table S2). As the bandwidth expanded, the effect size became smaller for inpatient healthcare utilization. Table S1 and Table S2 both show significant discontinuities at the statutory retirement age among women. These robustness checks further support our main results for women. Furthermore, we conducted a series of placebo tests using so-called falsified ages other than the statutory retirement ages. We found no consistent discontinuity in the outcomes on other ages for either gender (Additional file 1: Table S3). This further confirms the validity of the study design.

## Discussion

This study measures the causal effects of retirement on inpatient healthcare utilization in urban China by using a city-level sample including inpatient medical claims data and reimbursement records spanning 21 months. Our results provide evidence that retirement induced by the statutory retirement ages significantly decreased the overall use of inpatient healthcare in women. Meanwhile, no significant effects were found in men. This pattern of gender difference in retirement effects was robust when the sample was restricted to 10 years below and above the age threshold, or the bandwidth around the age threshold was changed. Our regression discontinuity results are consistent with several earlier studies that show a negative association between retirement and healthcare utilization [6, 27]. Further, the findings suggest that the effect of retirement is not the same for women and men. As the gender gap in statutory retirement ages is 10 years, this difference in retirement ages may be the source of the observed heterogeneity in the effects of retirement [16, 28]. In this regard, our results are broadly in line with a previous study, which found that early retirement leads to a decrease in hospitalization [29, 30].

Importantly, we find that the effect of retirement is actually the net result of two opposite effects: (1) an income effect, which reduces the intensive use of care (out-of-pocket expenditures and average length of stay), and (2) a substitution effect, which increases the extensive use of inpatient healthcare (hospital readmission and primary hospital visits). On one hand, there is a strong tendency for older adults to decrease out-of-pocket expenditures and reduce the length of hospital stays. This may be because older adults experience a substantial decrease in income, with a replacement rate of urban employees’ pension to average wages at around 44% in China [31]. Since primary hospitals tend to charge less for hospitalizations compared to higher level hospitals, the primary hospital visit ratio rises. This finding supports previous research suggesting a decrease in hospitalization after retirement for those with low socioeconomic status [32].

On the other hand, retirees are more likely to be hospitalized again once they have had at least one hospitalization during the past year. The increased reimbursement rate and reduced deductible of health insurance for retirees is a potential explanation. The findings also suggest that retirees go to a primary hospitals (i.e., community health center in urban areas) more often. In the 3-tier hospital system in China, primary hospitals have been serving as gatekeepers (rather than general practitioners) to preventive care, basic healthcare and rehabilitation services [33]. A similar conclusion suggests that the increase in hospital visits at retirement will be quite pronounced in a healthcare system without strong gatekeeper roles for general practitioners [34].

A key advantage of our medical claims data is that we can investigate the effects across groups that vary with disease category and type of service. The results suggest substantial disease heterogeneity in the effect of retirement on the overall use of inpatient healthcare utilization among women, with significant and releavant effects on NCD-related hospitalizations, but no such effects on hospitalizations due to communicable diseases and injuries. These findings indicate that, compared to women with other diseases, those with chronic conditions may have greater elasticity of demand for healthcare resources. In other words, women with chronic diseases have stronger incentives to adjust their healthcare utilization behavior after retirement, switching from inpatient to outpatient care or going to primary healthcare institutions more often.

Our results also show variation of effects type of service. For relatively low-cost services, such as nonoperative treatment, there were gains on the extensive margin, with an increase in hospital readmissions and the primary hospital visit ratio. For relatively highly invasive and expensive services, such as surgical treatment, retirement led to losses on the intensive margin, including a drop in out-of-pocket expenditures and length of stay. These effect heterogeneities are compatible to those of previous studies that report larger change for diseases that typically require highly specialized procedures [10, 35].

By providing a more nuanced explanation of how retirement affects the use of inpatient care in China, our study provides important insights theory and practice. First, we corroborate and extend the existing research on the effect of retirement on healthcare utilization, by applying Feldstein’s theoretical framework of demand for healthcare. Our work contributes to research on individual behavioral reactions to the institutional setting for retirement [36]. Second, our findings have practical value for the operation of tiered healthcare delivery in response to the growing demand for healthcare resources due to the increasing burden of NCDs in an aging society. In China, older adults are the primary driver of the substantial increase in healthcare utilization [37]. As hospitalization accounts for the majority of total health expenditures [38], understanding the factors that affect older adults’ inpatient healthcare utilization could provide valuable insights to diminish any unmet needs and ensure adequate provision of health services in China, while attaining health-related Sustainable Development Goals [39].

There are several limitations to our study. First, the age-based instrument for retirement might violate the exclusion restriction in a fuzzy regression discontinuity design. Although previous studies have used statutory retirement ages as instruments to estimate the consequences of retirement in China [9, 28, 40], recent research suggests that eligibility as a proxy for age is likely to be valid only for a small time interval around the statutory retirement ages [41]. This violation may occur in studies related to the health effects of retirement [41] or measuring age in years [42]. In our study, we thus focused on retirement effects on inpatient healthcare utilization and used age measured in months. Moreover, our robustness checks with intervals of 12 and 18 months around the statutory retirement ages provided consistent results. Second, the analysis presented here relies on the sample of inpatients during the pre-specified sample period. Thus, we should be cautious in generalizing the retirement effects that we have estimated to the total population. The object of our study was to examine the causal effect of retirement on the use of inpatient healthcare among those who had at least one hospitalization during the pre-specified time period. Finally, we did not add covariates to the estimations. However, under the assumption of the regression discontinuity design, it is not necessary to include them to obtain unbiased causal estimates [19]. To extend our findings, future research should establish the validity of our results in other contexts, elucidate further the underlying mechanisms generating the observed retirement effects on healthcare utilization, and measure retirement effects on other types of healthcare utilization.

## Conclusions

Retirement causes an 18% decrease in the overall use of inpatient healthcare in women, but does not affect healthcare utilization in men. The effect of retirement on inpatient healthcare utilization in women is the net result of two opposite effects: an increase in the extensive and a decrease in the intensive use of healthcare. These retirement effects vary with disease category and the type of service, with significant and relevant effects for NCD-related hospitalizations, but no such effects for hospitalizations related to communicable diseases or injuries. Policy makers considering raising the retirement age in China should also consider integrated healthcare delivery and precisely calibrated incentive schemes to ensure that older adults in China when raising the retirement age. The disease-related heterogeneous retirement effects that we identify may help developing appropriate health systems structures and incentive schemes to support better use of healthcare resources.

## Supplementary Information

**Additional file 1.**

## Data Availability

The dataset generated during and/ or analyzed during this study are available from the corresponding author on reasonable request.

## Abbreviations

NCD: Non-communicable disease
UEBMI: Urban Employee Basic Medical Insurance
ICD-10: International Classification of Disease 10th revision
