# Supplementary material for: The impact of retirement on inpatient healthcare utilization in Guangzhou, China: a regression discontinuity analysis of 189,031 health insurance claims
Source: BMC Geriatr. 2022 Apr 29;22:380. doi: 10.1186/s12877-021-02664-2 (PMC9052580; doi:10.1186/s12877-021-02664-2)
Supplement: Supplementary file 1 — Additional file 1. [file 12877_2021_2664_MOESM1_ESM.docx]

**Appendix Figure S1**: Density of Age Distribution to Test for Manipulation of the Assignment Variable

NOTE: The vertical lines at ages 60 for males and 50 for females are the statutory retirement ages.

| **Appendix Table S1**: Nonparametric Fuzzy RD Estimates with Restricted Sample (±10years) | | | | |  |  |
| --- | --- | --- | --- | --- | --- | --- |
|  | (1) | (2) | (3) | (4) | (5) | (6) |
|  | Total inpatient cost | Out-of-pocket expenditure | Hospital readmission | Average length of stay | Ratio of primary hospital visits | Ratio of tertiary hospital visits |
| *Panel A: Full sample* | | | | | | |
| Conventional | 14.88 | -21.59 | 0.037 | -0.34 | 0.05** | 0.01 |
|  | (-128.1 - 157.9) | (-62.38 - 19.19) | (-0.02 - 0.09) | (-1.30 - 0.61) | (0.01 - 0.09) | (-0.04 - 0.07) |
| Robust | 17.24 | -19.54 | 0.04 | -0.42 | 0.05** | 0.01 |
|  | (-146.1 - 180.5) | (-66.17 - 27.10) | (-0.02 - 0.10) | (-1.50 - 0.66) | (0.00 - 0.10) | (-0.05 - 0.07) |
| *Panel B: Male* | | | | | | |
| Conventional | 138.2 | 6.40 | -0.04 | 0.15 | 0.02 | 0.06 |
|  | (-63.16 - 339.5) | (-51.58 - 64.37) | (-0.13 - 0.04) | (-1.55 - 1.85) | (-0.04 - 0.08) | (-0.05 - 0.16) |
| Robust | 162.1 | 13.37 | -0.04 | 0.17 | 0.02 | 0.057 |
|  | (-65.43 - 389.6) | (-52.17 - 78.90) | (-0.13 - 0.05) | (-1.76 - 2.10) | (-0.05 - 0.09) | (-0.06 - 0.18) |
| *Panel C: Female* | | | | | | |
| Conventional | -90.54* | -45.69*** | 0.06** | -0.84* | 0.06*** | 0.00 |
|  | (-185.0 - 3.92) | (-73.55 - -17.84) | (0.00 - 0.12) | (-1.70 - 0.03) | (0.02 - 0.10) | (-0.06 - 0.06) |
| Robust | -102.7* | -47.94*** | 0.07* | -0.98* | 0.06*** | 0.00 |
|  | (-217.3 - 12.00) | (-81.86 - -14.03) | (-0.00 - 0.14) | (-2.00 - 0.05) | (0.02 - 0.11) | (-0.08 - 0.07) |
| NOTE: Entries in the first two rows in each panel are the conventional RD estimates with conventional variance estimators. Entries in the third and fourth rows in each panel are the robust RD estimates with robust variance estimators. 95% confidence intervals are in parentheses. *** p<0.01, ** p<0.05, * p<0.1. | | | | | | |

| **Appendix Table S2**: Nonparametric Fuzzy RD Estimates with Different Bandwidths | | | | |  |  |
| --- | --- | --- | --- | --- | --- | --- |
|  | (1) | (2) | (3) | (4) | (5) | (6) |
|  | Total inpatient cost | Out-of-pocket expenditure | Hospital readmission | Average length of stay | Ratio of primary hospital visits | Ratio of tertiary hospital visits |
| *Panel A: b = 12* | | | | | | |
| Full | 97.80 | -12.84 | 0.06 | -2.46 | 0.08 | -0.03 |
|  | (-232.7 - 428.3) | (-119.2 - 93.52) | (-0.12 - 0.24) | (-5.56 - 0.64) | (-0.03 - 0.18) | (-0.22 - 0.15) |
| Male | 450.2 | 50.99 | -0.01 | -0.33 | 0.04 | -0.07 |
|  | (-131.8 - 1,032) | (-132.1 - 234.1) | (-0.32 - 0.31) | (-5.38 - 4.73) | (-0.12 - 0.20) | (-0.34 - 0.21) |
| Female | -213.1* | -70.52* | 0.13* | -4.36*** | 0.11** | -0.03 |
|  | (-457.2 - 31.04) | (-149.8 - 8.78) | (-0.021 - 0.27) | (-6.96 - -1.77) | (0.02 - 0.21) | (-0.19 - 0.13) |
| *Panel B: b = 18* | | | | | | |
| Full | 29.73 | -18.52 | 0.06 | -1.62 | 0.05** | -0.01 |
|  | (-184.05 - 243.52) | (-84.31 - 47.26) | (-0.06 - 0.18) | (-3.65 - 0.41) | (0.00 - 0.10) | (-0.13 - 0.11) |
| Male | 290.60 | 42.39 | -0.03 | -0.35 | -0.01 | 0.02 |
|  | (-87.41 - 668.61) | (-69.42 - 154.21) | (-0.23 - 0.18) | (-3.68 - 2.99) | (-0.11 - 0.10) | (-0.16 - 0.20) |
| Female | -188.60** | -68.53*** | 0.14*** | -2.73*** | 0.10*** | -0.04 |
|  | (-350.62 - -26.59) | (-119.14 - -17.92) | (0.04 - 0.24) | (-4.50 - -0.96) | (0.04 - 0.16) | (-0.14 - 0.07) |
| *Panel C: b = 24* | | | | | | |
| Full | 23.63 | -16.85 | 0.0649 | -1.34* | 0.05* | 0.01 |
|  | (-142.2 - 189.5) | (-66.23 - 32.54) | (-0.028 - 0.16) | (-2.91 - 0.23) | (-0.00 - 0.11) | (-0.09 - 0.10) |
| Male | 252.9* | 35.42 | -0.02 | -0.36 | 0.01 | 0.07 |
|  | (-45.18 - 551.0) | (-49.41 - 120.3) | (-0.18 - 0.14) | (-2.98 - 2.25) | (-0.08 - 0.09) | (-0.08 - 0.21) |
| Female | -160.7* | -57.35* | 0.13** | -2.18** | 0.09** | -0.04 |
|  | (-350.7 - 29.31) | (-118.7 - 3.96) | (0.01 - 0.24) | (-4.20 - -0.17) | (0.02 - 0.16) | (-0.17 - 0.08) |
| *Panel D: b = 48* | | | | | | |
| Full | 16.43 | -25.39* | 0.04 | -0.42 | 0.05*** | 0.01 |
|  | (-82.51 - 115.4) | (-53.52 - 2.74) | (-0.02 - 0.09) | (-1.39 - 0.54) | (0.02 - 0.08) | (-0.05 - 0.06) |
| Male | 127.0 | -4.44 | -0.04 | 0.19 | 0.02 | 0.03 |
|  | (-47.09 - 301.0) | (-52.42 - 43.55) | (-0.13 - 0.06) | (-1.36 - 1.5) | (-0.02 - 0.07) | (-0.05 - 0.12) |
| Female | -101.1* | -47.44*** | 0.07** | -1.18* | 0.07*** | -0.01 |
|  | (-214.9 - 12.80) | (-81.97 - -12.90) | (0.00 - 0.15) | (-2.47 - 0.10) | (0.02 - 0.11) | (-0.09 - 0.07) |
| *Panel E: b = 72* | | | | | | |
| Full | 16.45 | -26.04** | 0.03 | -0.21 | 0.04*** | 0.02 |
|  | (-57.38 - 90.28) | (-46.48 - -5.59) | (-0.01 - 0.08) | (-0.96 - 0.54) | (0.01 - 0.06) | (-0.02 - 0.06) |
| Male | 93.93 | -13.28 | -0.04 | 0.23 | 0.02 | 0.04 |
|  | (-35.06 - 222.9) | (-47.83 - 21.26) | (-0.11 - 0.04) | (-0.96 - 1.43) | (-0.01 - 0.06) | (-0.02 - 0.11) |
| Female | -91.99** | -45.22*** | 0.06** | -0.97* | 0.05*** | 0.00 |
|  | (-176.4 - -7.52) | (-70.43 - -20.02) | (0.01 - 0.12) | (-1.97 - 0.03) | (0.01 - 0.09) | (-0.06 - 0.06) |
| NOTE: Entries are the robust RD estimates with robust variance estimators. 95% confidence intervals are in parentheses. *** p<0.01, ** p<0.05, * p<0.1. | | | | | | |

| **Appendix Table S3**: Placebo Age Tests | | | | | | |
| --- | --- | --- | --- | --- | --- | --- |
|  | (1) | (2) | (3) | (4) | (5) | (6) |
|  | Total inpatient cost | Out-of-pocket expenditure | Hospital readmission | Average length of stay | Ratio of primary hospital visits | Ratio of tertiary hospital visits |
| *Years from statutory retirement age* | | | | | | |
| *-3* |  |  |  |  |  |  |
| Full | 936.3 | 220.6 | 0.07 | -5.20 | 0.40 | -1.43 |
|  | (-2,965 - 4,837) | (-947.4 - 1,389) | (-2.26 - 2.40) | (-48.38 - 37.98) | (-1.60 - 2.40) | (-8.03 - 5.16) |
| Male | 901.7 | 209.4 | 0.06 | -12.63 | 0.18 | 0.05 |
|  | (-3,523 - 5,326) | (-1,336 - 1,754) | (-0.18 - 0.30) | (-55.01 - 29.76) | (-1.15 - 1.51) | (-0.18 - 0.27) |
| Female | 784.3 | 100.3 | 0.52 | 12.77 | 0.80 | -2.21 |
|  | (-2,978 - 4,547) | (-1,066 - 1,266) | (-2.32 - 3.36) | (-30.55 - 56.09) | (-1.21 - 2.80) | (-6.59 - 2.17) |
| *-2* |  |  |  |  |  |  |
| Full | -335.9 | 140.9 | -0.28 | -12.04 | -0.03 | -0.47 |
|  | (-3,606 - 2,935) | (-1,146 - 1,428) | (-1.86 - 1.30) | (-49.43 - 25.35) | (-0.77 - 0.72) | (-2.11 - 1.18) |
| Male | 75.78 | 312.9 | 0.35 | -13.70 | 0.13 | -0.17 |
|  | (-972.1 - 1,124) | (-995.8 - 1,622) | (-1.07 - 1.77) | (-45.14 - 17.74) | (-0.40 - 0.67) | (-1.19 - 0.84) |
| Female | -1.94 | -61.80 | -2.48 | -2.05 | 0.03 | -2.21 |
|  | (-5.90 - 2.01) | (-159.0 - 35.38) | (-6.29 - 1.34) | (-6.79 - 2.70) | (-0.60 - 0.66) | (-6.02 - 1.60) |
| *-1* |  |  |  |  |  |  |
| Full | -1,567 | -1,275 | -1.47 | -0.26 | -1.37 | 2.43 |
|  | (-8,799 - 5,664) | (-4,870 - 2,319) | (-7.05 - 4.12) | (-63.40 - 62.88) | (-5.15 - 2.41) | (-4.63 - 9.48) |
| Male | 1,151 | 243.6 | 0.90 | 7.60 | 0.06 | -0.09 |
|  | (-982.7 - 3,286) | (-248.8 - 736.0) | (-2.68 - 4.49) | (-5.02 - 20.23) | (-0.17 - 0.29) | (-1.0 - 0.81) |
| Female | 41.02 | -1,215 | 0.16 | 7.14 | 0.15 | -0.19 |
|  | (-2,907 - 2,989) | (-7,048 - 4,617) | (-0.82 - 1.12) | (-109.5 - 123.8) | (-0.23 - 0.54) | (-1.76 - 1.37) |
| *1* |  |  |  |  |  |  |
| Full | -170.9 | -81.37 | -0.020 | 2.43 | 0.32 | 0.11 |
|  | (-473.3 - 131.5) | (-223.0 - 59.33) | (-0.52 - 0.48) | (-4.45 - 9.30) | (-0.99 - 1.63) | (-0.09 - 0.31) |
| Male | -377.1 | -145.2 | 0.01 | -2.87 | 0.07 | 0.21 |
|  | (-1,127 - 373.1) | (-2,692 - 2,402) | (-0.41 - 0.43) | (-7.52 - 1.78) | (-0.18 - 0.32) | (-0.04 - 0.46) |
| Female | -48.76 | -16.98 | 1.18 | -2.44 | 0.51 | -0.10 |
|  | (-383.6 - 286.0) | (-86.09 - 52.13) | (-2.78 - 5.13) | (-5.82 - 0.95) | (-68.91 - 69.93) | (-0.34 - 0.14) |
| *2* |  |  |  |  |  |  |
| Full | 312.8 | 38.16 | -0.01 | 1.69 | 0.01 | -0.75 |
|  | (-215.9 - 841.5) | (-76.74 - 153.1) | (-0.19 - 0.18) | (-15.70 - 19.09) | (-0.08 - 0.11) | (-4.17 - 2.68) |
| Male | 1,599 | 451.0* | -0.05 | 6.72 | 0.00 | 0.04 |
|  | (-2,698 - 5,896) | (-65.34 - 967.3) | (-0.33 - 0.23) | (-4.20 - 17.64) | (-0.13 - 0.13) | (-0.27 - 0.35) |
| Female | -145.2 | -38.78 | -0.02 | -1.59 | -0.24 | 0.11 |
|  | (-3,018 - 2,728) | (-752.5 - 675.0) | (-0.43 - 0.39) | (-31.91 - 28.73) | (-1.06 - 0.58) | (-1.64 - 1.85) |
| *3* |  |  |  |  |  |  |
| Full | -261.6 | 4.237 | -0.88 | -7.72 | -2.23 | 1.93 |
|  | (-4,613 - 4,090) | (-1,210 - 1,219) | (-4.72 - 2.96) | (-81.03 - 65.59) | (-7.49 - 3.04) | (-3.03 - 6.90) |
| Male | -873.5 | 127.6 | -1.08 | 58.11 | -7.18 | 2.04 |
|  | (-10,881 - 9,134) | (-2,504 - 2,759) | (-6.90 - 4.75) | (-82.42 - 198.6) | (-20.20 - 5.84) | (-4.73 - 8.81) |
| Female | 1,390 | 375.4 | 1.07 | 7.53 | -0.33 | -0.07 |
|  | (-3,425 - 6,205) | (-917.6 - 1,668) | (-3.16 - 5.31) | (-6.81 - 21.86) | (-2.54 - 1.89) | (-2.59 - 2.45) |
| NOTE: Entries are the robust RD estimates with robust variance estimators. 95% confidence intervals are in parentheses. *** p<0.01, ** p<0.05, * p<0.1. | | | | | | |
